# A rapid evidence review on the effectiveness of institutional health partnerships

**DOI:** 10.1186/s12992-015-0133-9

**Published:** 2015-12-14

**Authors:** Ema Kelly, Vicki Doyle, David Weakliam, Yvonne Schönemann

**Affiliations:** Capacity Development International, Liverpool, UK; European ESTHER Alliance, Paris, France; Forum for Global Health, Dublin, Ireland; GIZ (The Deutsche Gesellschaft für Internationale Zusammenarbeit GmbH), Dublin, Ireland

**Keywords:** Effectiveness, Monitoring and evaluation, Development Cooperation, Partnership, Twinning, Institutional strengthening, Capacity development, Institutional Health Partnership, Global Health

## Abstract

**Background:**

Institutional Health Partnerships are long-term, institution to institution partnerships between high income and low and middle income countries which seek to build capacity and strengthen health institutions in order to improve health service delivery and outcomes. Funding for Institutional Health Partnerships has increased in recent years. This paper outlines a rapid evidence review on the effectiveness of this modality.

**Methods:**

A rapid evidence review of published and grey literature was conducted. Content relating to the effectiveness of working in partnership and methods and frameworks used were extracted and analysed. The results of this analysis were used to structure a discussion regarding the next steps to strengthen the evidence base for the effectiveness of institutional health partnerships.

**Results:**

The evidence review, including citation mapping, returned 27 published papers and 17 grey literature documents that met all of the inclusion criteria. Most of the literature did not meet the high standards of formal academic rigour and there was no original research amongst this literature that specifically addressed the effectiveness of institutional health partnerships. This was not surprising given institutional health partnerships do not lend themselves easily to case control studies and randomised control trials due to their high level of diversity and operation in complex social systems. There was, however, a body of practice based knowledge and experience.

**Conclusions:**

Evidence for the effectiveness of Institutional Health Partnerships is thin both in terms of quantity and academic rigour. There is a need to better define and differentiate Institutional Health Partnerships in order to measure and compare effectiveness across such a diverse group. Effectiveness needs to be measured at the level of individual partnerships, the bodies that facilitate partnership programmes and the level of health service delivery. There is a need to develop indicators and frameworks that specifically address the benefits and values of partnership working and how these relate to effectiveness. These indicators need to be content neutral of specific interventions which are already measured through routine project monitoring and evaluation. This will allow the development of methodological pathways to assess the effectiveness of institutional health partnerships. Until more primary research is conducted or published there is little benefit in further systematic reviews.

**Electronic supplementary material:**

The online version of this article (doi:10.1186/s12992-015-0133-9) contains supplementary material, which is available to authorized users.

## Introduction

This paper describes a rapid evidence review on the effectiveness of Institutional Health Partnerships (IHPs). The focus is on the quality and quantity of evidence for the effectiveness of IHPs and the methodological implications for future research. To the authors’ knowledge no previous reviews have been conducted in this specific area.

## Background

Efforts to strengthen health systems and attain better health outcomes in low and middle income countries (LMICs) are often hampered by health workforce issues whereby health workers have limited access to high quality education, mentoring support and continuing professional development opportunities [1, 2]. Health partnerships between institutions in the “global north” and low and middle-income countries (LMICs) seek to address this challenge through long term institution-institution partnerships that typically focus on capacity building, clinical service delivery and operational research. A central tenet of institutional health partnerships (IHPs) is the provision of long-term peer-peer support with the overall aim of strengthening the health workforce and its institutions.

There is a long history of institutional health partnership programmes between southern partners and those from Europe, North America and Canada. Working in partnership is now an established approach for North–south cooperation and achieving global health and development goals [3]. The Sustainable Development Goals (SDGs) give continued attention to North–South partnerships as a means of capacity building. In recent years there has been a renewed interest from governments, donors and other stakeholders in the potential opportunities and benefits that IHP programmes bring [4]. WHO recognises the contribution of institutional partnerships to health systems strengthening through “peer reviews, exchange visits, communities of practice, travelling seminars and institutional twinning” [5]. The Global Catalyst Group for Institutional Health Partnerships [3] was established in 2014 by WHO and other IHP programmes to promote the utility of institutional health partnerships in strengthening health systems and in delivering effective health services^1^.

Investment in institutional health partnership programmes has increased over the last decade, however, continued donor funding will necessitate establishing a stronger evidence base for their effectiveness. Whilst those who are engaged in partnership programmes believe that this approach is a valid, cost-effective and complementary form of technical cooperation [6] it is recognised that there is a lack of high quality evidence and inherent difficulties in measuring the effectiveness and benefits of partnership work [7]. To date, evaluations and research have largely focused on assessing activities and interventions of specific health partnerships rather than evaluating the effectiveness of the health partnership model, the added benefits of this approach or making comparisons with other forms of technical cooperation. Evidence is also needed about the role of IHPs within the development cooperation landscape and the added value of programmes dedicated to facilitating and supporting IHPs. It is therefore important to the partnership community to be able to demonstrate the effectiveness of IHPs and distinguish their niche role in the current era where funders and governments increasingly need to show results.

This paper reports on a rapid evidence review of the effectiveness of IHPs commissioned by the European ESTHER Alliance (EEA). The aim of this study was to review published and unpublished literature on the effectiveness of IHPs. The authors’ also draw on experiences in evaluating and facilitating IHP programmes as well as managing and evaluating large-scale international technical assistance programmes.

## Methods

A rapid evidence review of both peer-reviewed and grey literature was conducted between December 2014 and January 2015. Grey literature was included in this review because there are few published research papers in this area and unpublished documents and evaluations are likely to contribute to this emerging field. Inclusion criteria were agreed by the reviewers in consultation with the EEA Evidence and Effectiveness Working Group (experts in the field of IHPs). Criteria for inclusion were published or grey literature on IHPs where:

- the partnership is between northern and southern institutions (single or multiple);
- the partnership extends beyond a single project;
- the partnership undertakes activities with a health focus;
- the literature refers to effectiveness of partnership, not just the activities and/or intervention.

The two reviewers jointly assessed initial results of four articles to ensure consistent application of inclusion criteria and analysis and then divided the literature equally. Papers were only cross-checked when reviewers had doubts in relation to applying the inclusion criteria and/or analysis. Any disagreements were resolved by negotiation.

### Peer-reviewed literature

Two electronic databases were searched separately via OVID.MEDLINE and Web of Science, using a standard set of search terms with no year limits. Three categories separated by the Boolean operator “AND” were used:

1. **Institutional health partnership** (range of terms including health link, health partnership, hospital partnership, institutional partnership, paired partnership, institutional health partnership, twinning partnership, hospital twinning, collaborative link, collaborative partnership, North–south partnership).
2. **Geographical location** (developing country, low and middle income countries, Africa, Asia, Latin America).
3. **Effectiveness** (range of terms including effective, additional, benefit, evaluation, sustainability, ownership, flexibility, mutual, frontline, peer, cost, economic).

The titles and abstracts of all initial search results were screened and all articles unrelated to institutional health partnerships were excluded. All articles retained were then screened again to determine that they met the agreed inclusion criteria.

### Grey literature

The two reviewers were already familiar with much of the grey literature having conducted several evaluations of IHP programmes since 2012. European ESTHER Alliance members also supplemented the grey literature with documents relating to formal evaluations of individual IHPs or partnership programmes as well as any other documents identified as relating to effectiveness of IHPs (eg unpublished research, think pieces, case studies, conference presentations).

### Final selection and citation mapping

Following collation of all documents that met the inclusion criteria, and after removing all duplications, bibliographies were reviewed and references that were of potential relevance were assessed against the inclusion criteria.

### Evidence assessment

All documents were assessed for the level of evidence they provided. The traditional hierarchy of evidence scoring was not used due to the limited amount of original research conducted in this field. IHPs often aim to make improvements to institutions and their workforce which are complex, change over time and are context specific. These kinds of intervention do not lend themselves easily to the types of research that are at the top of the evidence hierarchy such as randomised controlled trials. Therefore an adapted scoring system [8] was used to more easily distinguish between the evidence included in this review.

- **Level 0:** Expert opinion/advocacy.
- **Level 1:** Coherent description of what was done and with clear rationale (logical and convincing).
- **Level 2:** Includes data that shows change, but attribution not proven.
- **Level 3:** Demonstrate causality through use of control or comparison group.
- **Level 4:** At least one replication studied independently (shows repeatability).
- **Level 5**: Systematic review.

### Data analysis

The analysis of literature was “content neutral” with respect to specific interventions, since these are rarely comparable and specific educational, clinical, and management interventions are already well documented in the international scientific literature. All published papers and short (<15 pages) grey literature documents were scanned in their entirety. For longer documents (mainly programme evaluations), initially the executive summary was reviewed and then only relevant other sections of the document. Short summaries were produced for each document reviewed and key data was extracted.

## Results

### Quality and quantity of evidence

For the database searches (no year limits), MEDLINE produced 49 hits and Web of Science 98 hits. After excluding those that did not meet inclusion criteria and deleting duplicates, 18 published journal articles were included from the initial database search. An additional nine published articles were included after citation searching and review of documents supplied by EEA members.

The grey literature search returned a total of 42 documents. These were retrieved from the reviewer’s previous work on IHPs and supplied by EEA members from Ireland, France, Germany, Norway and UK. After excluding those that did not meet inclusion criteria and after deleting duplicates, a total of 17 grey literature documents were included.

Therefore a total number of 44 published and grey literature documents were included in the review; of this 27 articles were from peer-reviewed literature and 17 from grey literature. Table 1 shows the number, type of documents and level of evidence using the previously cited assessment criteria.

**Table 1:** Definitions or descriptions of IHPs from the three systematic reviews included in this rapid review

Health links are long term partnerships between UK health institutions and their counterparts in developing countries. …. Links are typically small partnerships that work in areas such as capacity building or clinical service delivery. Whereas some links are set up as small charities with expenses covered by the individuals involved, others are funded directly by the NHS. Ultimately, one of the main objectives of health links is to improve the health of the population in the corresponding developing country.”	… international partnerships, … lead, stimulate, and facilitate action on health challenges through programming, advocacy and technical support. …. Partners increasingly seek mutuality of benefits, including two way flow of energies, expertise and knowledge to justify investment.”	Partnerships to share learning and resources between UK institutions and collaborators in Low and Lower Middle Income Countries are one model to improve health care delivery. It has been proposed that such links promote genuine understanding and respect for different societies and cultures, offer a more sustainable, locally led model of development, build capacity and strengthen health systems in developing countries.”
Smith [9]	Syed et al. [1]	Jones et al. [1]

The full list of papers reviewed and their level of evidence categorisation is available in Additional file 1.

Excluding editorials, 63 % of the journal articles were written by those directly involved in implementing the IHP. Fifty-five per cent of journal articles and grey literature, excluding editorials, reported on multiple partnerships. Sixty-eight per cent of the literature reviewed was categorised as Level 1 or Level 2 evidence. The literature at Level 0 (n = 10) showed an overarching support for the IHP approach and a belief that this type of technical cooperation brings a wide range of benefits to both northern and southern institutions and potentially strengthens health systems.

The literature at Levels 1 and 2 were case studies, evaluations and research papers/reports. The case studies (n = 12) represent a valuable source of practice-based knowledge with good descriptions of the process of implementation but largely limited to activity and output reporting. Whilst the Level 2 case studies report change and present higher levels of analysis, the evidentiary value on the effectiveness of IHPs is limited and attribution of change unproven. Case studies included a self-reported outline of surgical capacity building in Uganda which demonstrated long term effects through having a robust baseline and subsequent collection and analysis of indicators [9]. Corbin et al. [10] used the Bergen Model of Collaborative Functioning (BMCF) to map the successes and failures of one organisation’s North–south partnership experience. Reflecting on a ten-year partnership between the UK and Swaziland in public health, Wright et al. [11] identify six principles behind their success and report on quantified improvements in TB and epilepsy indicators demonstrated through RCTs.

The evaluations (n = 10) were largely externally commissioned and primarily reliant on secondary data supplemented with qualitative primary data. In general these were comprehensive evaluations of IHP programmes with clearly defined methodologies and greater depth and quality of analysis, however, only half of the evaluations reported the use of an evaluation framework. Paterson & Telykov [12], Bouscharain & Moreau [13] and Doyle & Kelly [6, 14] in evaluating partnership programmes all used either the OECD/DAC evaluation criteria for development programmes or a logic model framework. All evaluations reviewed were rated at Level 2 in that the attribution of change is not proven.

Of the seven research papers/reports reviewed all were rated at Level 2, none of which specifically focused on the effectiveness of the IHP approach. Several studies looked at benefits of partnership working to the developed country partner through competency development or reverse innovation. Smith et al. [15], Kiernan et al. [16] and Longstaff [17] mapped skills gained through international work onto NHS leadership and competency frameworks. Busse et al. [18] used the Association of Schools and Programs of Public Health global health competency framework. Hagen et al. [19] conducted a phenomenological/hermeneutical study to look at the development of cultural competence through exchange.

The three systematic reviews, categorised as Level 5, recognised that the current standard of literature on which their reviews were based did not meet the high standards of formal academic rigour with little published or unpublished literature on the specific area they were reviewing in relation to IHPs. The particular focus of the three systematic reviews were:

- Health outcomes (Smith [20]): concludes that there is a lack of high quality research in this area but that broad trends appear to demonstrate improved health outcomes as a consequence of health links.
- Reverse innovation (Syed et al. [21]): concludes that benefits were largely soft (employee morale, learning, better information sharing, personal development, improved patient relationships). The study did not find evidence for the broader ‘impact’ of these benefits on health systems.
- Benefits to UK partners (Jones et al. [22]): concluded that there is little published or unpublished literature on the impact of volunteering and the existing evidence base was primarily descriptive. The review identified six domains of individual benefit to the UK and adapted them into an existing framework (developed by Wales for Africa) to show how the experience of overseas volunteering could impact on individuals, institutions and health care services.

There was no original research that specifically addressed the effectiveness of IHPs. A previous review of partnerships across all sectors [23] identifies that there are very few detailed and theoretically grounded case studies of partnerships with most research based on secondary data, questionnaire surveys or personal impressions.

The literature was also thin in terms of describing methods, indicators and frameworks for measuring the effectiveness of IHPs. The three systematic reviews identified an urgent need for more rigorous and standardised methods and tools for reporting costs, benefits, effectiveness, outcomes and impact of IHPs.

## Discussion

### Strengths and limitations of the review

This review is the first seeking to identify the quantity and quality of evidence on the effectiveness of IHPs. This is not a full systematic review, but we sought to use the highest levels of rigour possible given the short time frame available for the work. Robust methods were used to review current grey and published literature. It is possible that key documents may have been missed due to the more limited search strategy employed. Much of the literature reviewed did not specifically evaluate the IHP model, but alluded to the benefits and effectiveness of using a partnership approach.

### Main findings of the review

Overall the review identified the thinness of the evidence base in terms of the quantity of studies and their location at the lower end of the evidence scale. The vast majority (93 %) of the documents reviewed were Level 0, 1 or 2. These results are not surprising given that IHPs do not lend themselves easily to case control studies and randomised control trials [24], which sit at the top end of the evidence hierarchy. IHPs seek to make improvements to institutions and their workforce .  These changes are complex, evolve over time and are context specific. In addition, attribution is notoriously difficult to prove in settings with a myriad of partnerships and projects overlapping and interacting [7].

Limitations from the literature reviewed included; small sample size, self-reported evaluations, lack of baselines and measurement of activities and outputs rather than outcomes and impact. No studies used control groups, comparison groups or tested for repeatability.

The need to develop rigorous and standardised methods and tools for measuring the effectiveness of partnerships was identified in the three systematic reviews and is also a clear conclusion from this review.

There are, however, a number of conceptual questions that need to be addressed before such frameworks and methods can be developed. These relate to the definition and differentiation of IHPs and defining what is meant by effectiveness of IHPs. This discussion outlines these questions building on the analysis of the methods and frameworks (or lack thereof) reviewed from the literature.

### Implications for future monitoring and evaluation and research

#### Definition and differentiation

The word partnership is both overused and misused. Distinguishing IHPs from other forms of technical cooperation is not necessarily straightforward. The majority of documents did not provide a definition of IHPs. The definitions provided in the systematic reviews are broad descriptors of IHPs and do not clearly define their specific and essential characteristics, see Table 2.

**Table 2:** Level of evidence and type of document reviewed

	Level 0	Level 1	Level 2	Level 3	Level 4	Level 5	Total
Grey literature	2	1	14	0	0	0	17
Journal article	8	6	10	0	0	3	27
Total	10	7	24	0	0	3	44

The various definitions have some common elements: institution-to-institution partnerships, relationship between low income and high-income settings and capacity development. Many of the IHP programme facilitators have listed definitions on their websites or educational materials. For example the Tropical Health and Education Trust (THET) define IHPs in the UK as *“long-term partnerships between UK health institutions and their counterparts in developing countries. Partnerships aim to improve health services in developing countries through the reciprocal exchange of skills, knowledge and experience between partners in the UK and those overseas”* [25].

The ultimate aim of partnerships is variously defined as: improving health outcomes; improving health service delivery; strengthening the health workforce and strengthening health systems. The documents reviewed and key proponents of partnership working also identify a number of benefits that arise from a value-led partnership approach and these often include: mutual benefit; local ownership; flexibility; access to frontline services; peer-to-peer support and long-term commitment. Whilst there is a set of values shared by the partnership movement they are not exclusive to it. The multiplicity of aims, scale and technical focus creates a challenge for creating a standard framework or generic indicators that can be used to measure and compare effectiveness and benefits of working in partnership. This creates difficulties in assessing effectiveness particularly in comparison to other forms of technical cooperation.

A further layer of complexity is that health partnerships operate at community, primary, secondary, tertiary and national levels within the health sector. IHPs work within single regions, single countries or across countries and continents. The focus of IHPs varies from those with a narrow technical or clinical focus to those with a broad institutional or health system remit. Partnerships also vary by their stage of development from the first steps being taken towards partnership to a maturity based on many years collaboration. Hence when looking at effectiveness there is an intrinsic problem of being able to compare like with like within such a differentiated field. Defining and differentiating institutional health partnerships in terms of their scale, scope and purpose is a vital first step in being able to develop methodological pathways for assessing the effectiveness of partnerships beyond their own project log frames.

### What do we mean by effectiveness?

The Oxford English Dictionary defines effective as: “powerful in effect; producing a notable effect”. The OECD/DAC Evaluation Framework for Evaluating Development Assistance [26] defines effectiveness as: “a measure of the extent to which an aid activity attains its objectives.” Hence effectiveness needs to show measureable change against a specified objective. This is not only at the level of individual partnerships, but as Horton et al. [23] proposed in their analysis of partnership knowledge and practice, effectiveness should be assessed at three levels:

- the level of individual partnerships;
- the level of organisations that facilitate and manage a portfolio of partnerships;
- the level of health service delivery and systems.

### The effectiveness of individual partnerships

The objectives of individual partnerships usually relate to improving health service delivery within a specific institution or institutions and ultimately improving health outcomes for users of those services. Partnerships that are funded externally will usually require monitoring and evaluation (M&E) of project outputs and where possible outcomes. However, partnerships typically have modest resources and expertise to undertake M&E and existing information systems within LMIC institutions are often weak. Monitoring is often limited to quantifying activities and outputs, such as number of personnel trained or services provided, with improvements in quality of care or other outcomes rarely measured. The journal articles relating to individual IHPs which were categorised as Level 2 in this review go beyond measuring activities and outputs but they are still relatively few in number (see Additional file 1).

For similar reasons M&E rarely continues beyond the envelope of project funding. This makes it difficult to assess the effectiveness of partnerships in embedding and sustaining changes within institutions. Ensuring sustainable change is notoriously difficult due to many health system related factors including staff turnover, weak supervision, supply chain problems and financial resourcing [27]. The fact that IHPs are built on a long-term commitment implies that they should be able to sustain change beyond project funding. Evidence of this would be of key interest to donors and Ministries of Health and would require monitoring and evaluation to occur beyond the project-funding envelope.

Monitoring and evaluation in IHPs primarily focuses on project outputs and outcomes and rarely measures the process or additional benefits gained through working in partnership. There is increasing interest in the partnership movement in defining what makes a quality partnership; this is a move to focus on process as well as the results of partnership activities. There are a number of tools that have recently been developed to assess adherence to quality of partnership standards [28, 29]. Currently, these quality of partnership standards are based on practice-based knowledge and there is a lack of an evidentiary base of how they relate to effectiveness. If a partnership delivers successful change within an organisation or service does that make it an effective partnership? If a partnership is deemed to be high quality does that mean that it is effective in delivering change within organisations and services? Quality of partnership is linked to the values that underpin the partnership movement. Further work is needed to understand:

- how these values and standards relate to effectiveness;
- to what extent they are best practice in development cooperation or reflect something specific to the partnership model;
- and to what extent they link to some of the additional benefits ascribed to working in partnership.

In this review a number of studies have been undertaken to assess some of the additional benefits ascribed to partnership working. In particular, building professional competencies in northern partner institutions was the topic of some of the more methodologically robust studies in this review [15–18].

Hence there are three levels at which individual health partnership effectiveness is being assessed.

- The intervention or activities undertaken within the partnership through monitoring and evaluation.
- The quality of the partnership through adherence to principles or charters and increasingly through self-assessment tools.
- The degree to which the partnership has delivered additional benefits beyond the project log frame such as sustainability or mutual benefit, usually through research studies.

At the first level it is almost impossible to compare IHPs due to the diversity of objectives, activities and scale. However, it should be possible to collate a set of indicators based on research to look at both the quality of partnership and additional benefits of working in partnership. These indicators could be used consistently across a number of IHPs to build a better evidence base and conceptual framework linking the partnership process to partnership benefits. This would have the dual benefit of providing evidence of the benefit of working in partnership and being able to distinguish effective from non-effective partnerships. These partnership indicators would complement standard M&E that measures the effectiveness of activities and interventions being delivered by the partnership. Providing robust evidence of partnership benefits and their link to the partnership process would enable the partnership movement to move from faith to science.

### The effectiveness of organisations that facilitate and manage a portfolio of institutional health partnerships

The effectiveness of IHPs is also influenced by the support and guidance provided by the body facilitating the partnership (eg ESTHER national programmes, THET, Wales for Africa and the American International Health Alliance (AIHA)). In particular, the facilitating body may assist partnerships in addressing knowledge and skills gaps in relation to project management, development cooperation, cultural competence and monitoring and evaluation. The effectiveness of the facilitating body in meeting these needs should have an impact on the effectiveness of the partnerships they facilitate.

Networking is a further benefit offered by the facilitating body. For example, the EEA connects its member countries and the individual IHPs within their programmes, leading to opportunities for coordination and collaboration at country and regional levels. Through the EEA, links can be established with donors, WHO and other organisations involved with IHPs. Implementing partners are connected with national governments and donor programmes within the countries. The networking approach facilitates scaling up of initiatives, as seen in the spread strategy of the WHO African Partnerships for Patient Safety. These various aspects of networking provide additional support for IHPs through information exchange, learning possibilities, joint activities (pooling funds), scaling up outcomes and possibilities of additional funding.

Facilitating bodies and their partnership programmes are usually regularly evaluated against the objectives set with the donor for the overall programme of work. This review included a number of evaluations of facilitating bodies or partnership programmes: however, these focused on the effectiveness of implementation without necessarily analysing the underlying strategy of the programme. They also highlighted the varied scale and technical focus of IHP programmes as well as the wide range of approaches taken by facilitating bodies in providing support to IHPs.

The facilitating body, often with funders, sets the criteria for providing funding to IHPs. This shapes the geographical and technical focus of funded partnerships, their scale and quantity. Underlying this, often implicitly, is a perspective on the role of IHPs within the array of development cooperation modalities used by bilateral donors. In most countries the amount of funding given to IHPs is small in comparison to other funding in international health through multilateral agencies, technical assistance and research.

There are three levels at which the effectiveness of the facilitating bodies of partnerships programmes should be assessed.

- Their effectiveness at providing guidance and support to individual IHPs particularly in those areas which may be outside their usual professional expertise.
- Their effectiveness at creating and strengthening networks for coordination, collaboration and funding at national and international levels.
- Their effectiveness at providing a portfolio of IHPs that ultimately meets the donor objectives for this mode of development cooperation.

This third aspect of effectiveness requires clarity of the niche role of IHPs in the development cooperation landscape to improve health service delivery and systems in low and middle-income countries. In the literature reviewed, none of the papers clearly identify the specific niche role of IHPs within the wider international development cooperation landscape.

### The effectiveness of IHPs in improving health service delivery and systems

There is also a lack of evidence of how IHPs are best utilised within the wider development efforts to improve health outcomes. There are a number of roles that have been identified as being suited to IHPs:

- health workforce strengthening;
- institutional strengthening;
- peer mentoring;
- innovation;
- supporting frontline services;
- supporting clinical areas which traditionally do not get much attention from donors eg epilepsy, non-communicable diseases, surgery, mental health.

**Fig. 1:**
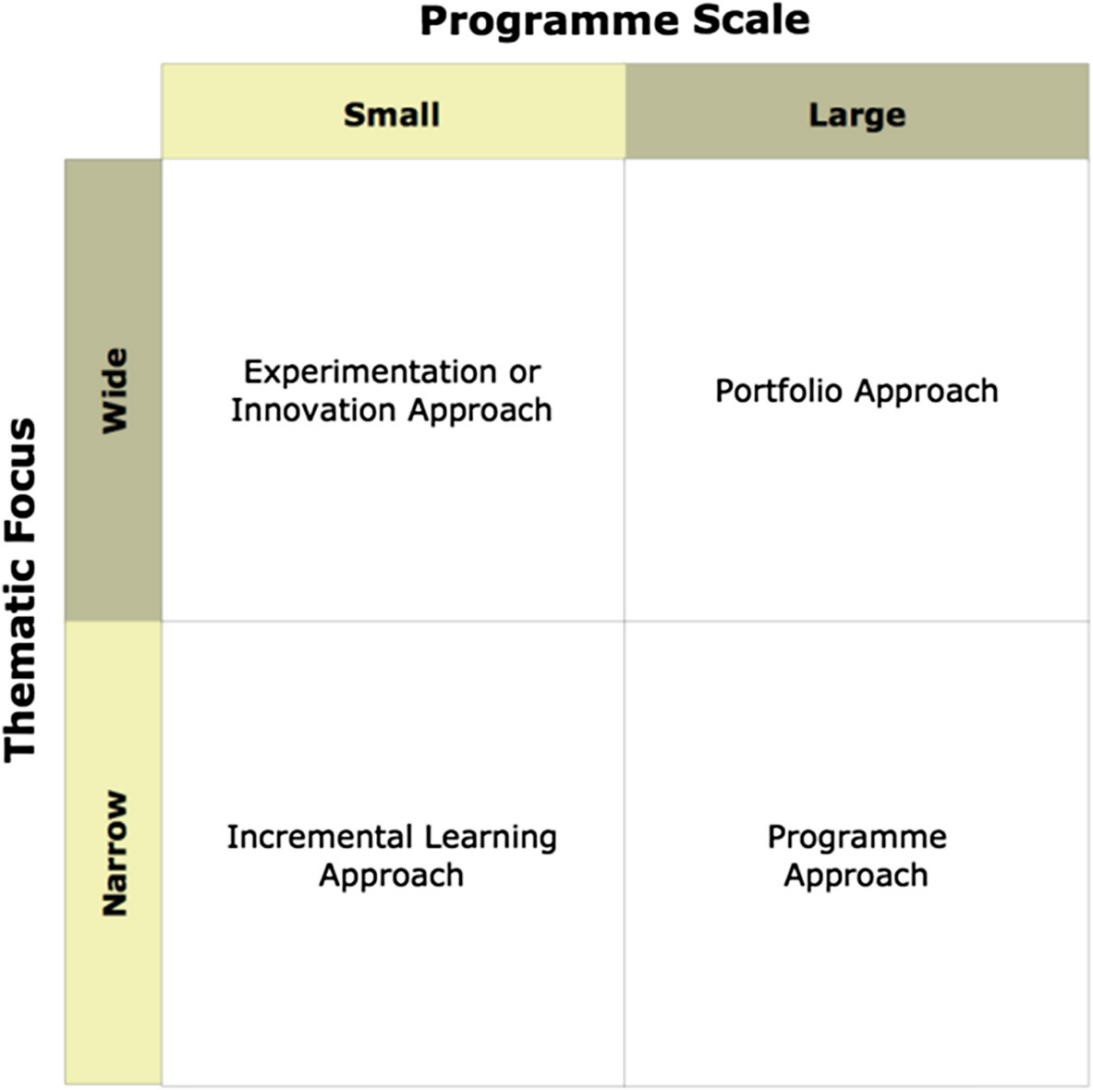
Categorisation of partnership portfolios by scale and thematic focus [6]

The evaluation of the European ESTHER Alliance proposed a differentiation of roles for IHP programmes (see Fig. 1) within development cooperation dependent on scale and technical focus [6]; with small-scale programmes being best suited for experimentation or incremental learning. This implies that the facilitating body needs to attach importance to dissemination of lesson learning if the partnership programme is going to demonstrate measurable change within a health service or system. Large programmes with a narrow thematic focus can take a programmatic approach similar to large technical assistance programmes that are narrowly focussed. Large programmes with a wide thematic approach have the opportunity to work in any of these ways or a combination of them; however there is a risk that a *scattergun approach* will result in improvements in individual institutions but without measureable changes within the health system.

One of the challenges in looking at the effectiveness of IHPs at the service delivery and system level is that of scale and coverage. Only a small percentage of institutions working in a thematic area may have partnerships. Some IHPs have chosen to be strategic in their choice of partner, linking to institutions that provide education across a whole country or countries or linking to institutions with national or regional reach. The WHO APPS movement provides an example of how successful partnership initiatives can be encouraged to be taken up by a wider movement. But for many institution-to-institution partnerships, dissemination and scale up of successful interventions is beyond the scope of their partnership activities. In order to be able to measure the effectiveness of IHPs there needs to be clarity on how the work of individual IHPs working at an institutional level can impact on health service s and the wider system. The literature is increasingly showing that spread, scale up and dissemination do not happen unless they are planned and resourced [30]. This continues to be an important consideration for donors and facilitating bodies when designing IHP programmes.

The scale and coverage of IHP portfolios also effects their ability to adhere to the aid effectiveness agenda. The partnership movement should continue to be based on values of local ownership and alignment; however the issues of fragmentation and harmonisation with other development activities are challenging to the smaller scale non-programmatic IHPs. For a beneficiary country Ministry of Health, it can be extremely challenging to keep track of multiple partnerships and projects active within the health sector, carrying the risk of losing out on good practice, lesson learning and the potential to go to scale. Facilitating bodies (and donors) have a potentially important role in facilitating communication of best practice and lessons learned particularly with partner country governments. Best practices from technical cooperation programmes would indicate that individual partnerships should seek opportunities to link with other projects and activities and build communication and dissemination into their plans from the start. Measuring the effectiveness of these vital spread and dissemination activities is challenging.

## Conclusion

Many modalities used within technical cooperation between high income and low and middle income countries lack a robust evidence base due to the methodological difficulties inherent in comparing interventions made within real world complex social systems. Clinical interventions can and should be based upon robust evidence at the top of the evidence hierarchy such as randomised control trials. By contrast the modality through which capacity and institutional strengthening occurs within health systems and institutions may need to build its evidence base using other forms of robust methods from areas such as management science, implementation science and social science. Institutional Health Partnerships are being increasingly promoted as a promising approach for strengthening the health workforce and health systems. However, evidence is currently thin and there are few frameworks and indicators specific to the effectiveness and benefits of working in partnership. Until further primary research findings are published or concluded there is little merit in conducting further systematic reviews within the field due to the paucity of robust evidence. A much needed first step is, therefore, building a clear conceptual framework that defines and differentiates IHPs and starts to build indicators and models linking the values underpinning partnership working to their benefits and effectiveness in institutional strengthening and capacity building along with identifying their niche within the development cooperation field. This paper proposes a number of levels at which effectiveness of IHPs should be assessed: at the level of individual partnerships; at the facilitating body level; and health service delivery or system levels. There are also three facets important in assessing individual health partnership effectiveness: the intervention or activities undertaken within the partnership; the quality of the partnership; and the degree to which the partnership has delivered additional benefits beyond the project. Each of these levels requires its own methodological pathways focusing on measuring change, with an ultimate goal of being able to undertake studies comparing IHPs and the IHP modality to other forms of technical cooperation.

### Addendum

As identified in the limitations this was a rapid evidence review. One paper that was not identified in the search but would have been useful to the review was identified by the peer reviewers of this paper. Rutter et al. [31] describes a participatory process to develop an evaluation framework for patient safety partnerships giving indicators not only for the effectiveness of the patient safety interventions but also partnership strength and national spread. The indicators for partnership strength were based on practice based knowledge and expert review and were not validated. Indicators for national spread were activity based. The framework Rutter et al. develop supports the use of indicators to measure partnership effectiveness and spread within health systems as well as intervention effectiveness and is a useful step forward in the development of context appropriate evaluation frameworks.

## Additional file

Additional file 1:
**List of all literature included in the review outlined in this paper.** Includes a table listing the papers by level of evidence and an additional table outlining additional benefits of partnership cited in the papers. (DOCX 33 kb)

## Footnotes

1 Members of Global Catalyst Group for institutional health partnerships: WHO African Partnerships for Patient Safety, European ESTHER Alliance, International Hospital Federation, THET, American College of Health Care Executives
