# Erratum to: A rapid evidence review on the effectiveness of institutional health partnerships

**DOI:** 10.1186/s12992-016-0140-5

**Published:** 2016-01-21

**Authors:** E. Kelly, V. Doyle, D. Weakliam, Y. Schönemann

**Affiliations:** Capacity Development International, Liverpool, UK; European ESTHER Alliance, Paris, France; Forum for Global Health, Dublin, Ireland; GIZ (The Deutsche Gesellschaft für Internationale Zusammenarbeit GmbH), Dublin, Ireland

It has come the publisher’s attention that the original version of this article [1] unfortunately published Tables 1 and 2 in the incorrect order. All references to the tables in the main body of the text were correct in the original article. The tables have been updated in the original version of this article accordingly, and published in this Erratum for quick reference.

**Table 1:** Level of evidence and type of document reviewed

	Level 0	Level 1	Level 2	Level 3	Level 4	Level 5	Total
Grey literature	2	1	14	0	0	0	17
Journal article	8	6	10	0	0	3	27
Total	10	7	24	0	0	3	44

**Table 2:** Definitions or descriptions of IHPs from the three systematic reviews included in this rapid review

Health links are long term partnerships between UK health institutions and their counterparts in developing countries. …. Links are typically small partnerships that work in areas such as capacity building or clinical service delivery. Whereas some links are set up as small charities with expenses covered by the individuals involved, others are funded directly by the NHS. Ultimately, one of the main objectives of health links is to improve the health of the population in the corresponding developing country.”	… international partnerships, … lead, stimulate, and facilitate action on health challenges through programming, advocacy and technical support. …. Partners increasingly seek mutuality of benefits, including two way flow of energies, expertise and knowledge to justify investment.”	Partnerships to share learning and resources between UK institutions and collaborators in Low and Lower Middle Income Countries are one model to improve health care delivery. It has been proposed that such links promote genuine understanding and respect for different societies and cultures, offer a more sustainable, locally led model of development, build capacity and strengthen health systems in developing countries.”
Smith [2]	Syed et al. [3]	Jones et al. [3]

A slight change in the author details section of the PDF was also made with this Erratum. The 4^th^ affiliation incorrectly read: GIZ (The Deutsche Gesellschaft für Internationale Zusammenarbeit GmbH), Dublin, Ireland. In the original article, the city and country have been amended to read: Berlin, Germany.
