# Supplementary material for: A rapid evidence review on the effectiveness of institutional health partnerships
Source: Global Health. 2015 Dec 14;11:48. doi: 10.1186/s12992-015-0133-9 (PMC4678480; doi:10.1186/s12992-015-0133-9)
Supplement: Additional file 1: — List of all literature included in the review outlined in this paper. Includes a table listing the papers by level of evidence and an additional table outlining additional benefits of partnership cited in the papers. (DOCX 33 kb) [file 12992_2015_133_MOESM1_ESM.docx]

# Documents reviewed for evidence on IHPs

**Alphabetical full list of references provided below the table**

| **Level 0** | **Grey Literature/Published Reports**  Crisp 2007  Rao 2013  **Journal Article: Editorial/think piece/commentary**  Costello et al 2000  Crisp 2014  Easterbrook 2011  Leather et al 2010  Parry & Percy 2007  Ritman & Zegeye 2012  Roodenbeke 1994  Smith 2012 |
| --- | --- |
| **Level 1** | **Grey Literature/Published Reports**  COPP (undated)  **Journal Article: Case Study**  Amde et al 2013  Busse et al 2013  Cadee et al 2013  Kinnear et al 2013  Tierney et al 2013  **Journal Article: Review**  Hopkins et al 2013 |
| **Level 2** | **Grey Literature/Published reports: Case Studies/Research**  Abualela 2014  ESTHER France 2012 x 3  Longstaff 2010  **Grey Literature/Published reports: Evaluations and reports**  Bouscharain & Moreau 2012  Doyle & Kelly 2012  Doyle & Kelly 2013  Haarberg et al 2011  Paterson et al 2007  Schönemann & Weinmann 2010  Thomas et al 2011  THET 2014  **Journal Article: Case Study**  Corbin et al 2013  Haglund et al 2011  Wright et a 2010  **Journal Article: Evaluation/Research**  Baguley et al 2006  Beran 2010  Busse et al 2014  Hagen et al 2009  Haugland et al 2013  Kiernan et al 2014  Smith et al 2012 |
| **Level 5** | Jones et al 2013  Smith 2013  Syed 2012 |
| **Identified after review** | Rutter et al 2014 |

Abualela N (2014) "An Evaluation of Selected Multi-Country Partnerships".  Submitted in partial fulfillment of the requirements for the award of Master’s in International Public Health, Liverpool School of Tropical Medicine.

Amde, W.K., Sanders, D. & Lehmann, U., 2014. Building capacity to develop an African teaching platform on health workforce development: a collaborative initiative of universities from four sub Saharan countries. Human resources for health, 12(1), p.31.

Baguley, D., Killeen, T., J., Wright, J., (2006). International health links: an evaluation of partnerships between health-care organizations in the UK and developing countries, *TROPICAL DOCTOR* 2006; **36:**149-154.

Beran, D., Silva Matos, C., & Yudkin, J. S. (2010). The Diabetes UK Mozambique Twinning Programme. Results of improvements in diabetes care in Mozambique: a reassessment 6 years later using the Rapid Assessment Protocol for Insulin Access. *Diabetic Medicine*, *27*(8), 855–861.

Bouscharain, G and Moreau, J-B, 2012,Evaluation of the Institutional Twinning Instrument in the Countries covered by the European Neighbourhood Policy.  Final Report HTSPE.

Busse, H., Aboneh, E. & Tefera, G., 2014. Learning from developing countries in strengthening health systems: an evaluation of personal and professional impact among global health volunteers at Addis Ababa University¿s Tikur Anbessa Specialized Hospital (Ethiopia). Globalization and health, 10(1), p.64.

Busse, H., Azazh, A., Teklu, S., Tupesis, J. P., Woldetsadik, A., Wubben, R. J., & Tefera, G. (2013). Creating Change Through Collaboration: A Twinning Partnership to Strengthen Emergency Medicine at Addis Ababa University/Tikur Anbessa Specialized Hospital-A Model for International Medical Education Partnerships. *Academic Emergency Medicine*, *20*(12), 1310–1318.

Cadée, F, Perdok, H, Sam, B, de Geus, M Kweekel, L, 2013. “Twin2twin” an innovative method of empowering midwives to strengthen their professional midwifery organisations. *Midwifery*, 29(10), pp.1145–50.

COPP, Upgrading health systems to reduce maternal and perinatal mortality in the largest maternity hospital in Africa: a HSE-Irish Aid collaboration. Poster Presentation, Patient Safety Meeting

Corbin, J. H., Mittelmark, M. B., & Lie, G. T. (2013). Mapping synergy and antagony in North-South partnerships for health: a case study of the Tanzanian women's NGO KIWAKKUKI. *Health Promotion International*, *28*(1), 51–60.

Costello A, Zumla A. Moving to research partnerships in developing countries. BMJ 2000;321:827–9

Crisp, N., 2014. Mutual learning and reverse innovation--where next? Globalization and health, 10(1), p.14.

Crisp, N 2007 Global Health Partnerships: The UK contribution to health in developing countries, COI

Doyle, V and Kelly, E, 2013.  European ESTHER ALLIANCE: Study. Evaluation Report, Capacity Development International

Doyle, V and Kelly, E, 2012, International Health Links Funding Scheme Evaluation on behalf of THET, Capacity Development International

Easterbrook, P.J., 2011. Institutional partnerships in global health. Clinical Medicine, 11(2), pp.112–113.

Esther France (2012) Three Exemplary Projects.  "Decentralisation of HIV/AIDS care through mentoring in the Central Region of Cameroon."

Esther France 2012, Three Exemplary Projects. "Strengthening Care for People Living with HIV in Abidjan Central Prison (MACA)"

Esther France 2012, Three Exemplary Projects.   "Nouygal" Project: Care and follow-up of HIV-infected pregnant women at Bamako's Gabriel Toure Hospital.

Haaberg, K, Dale, E, Whist, E 2011 Review of the Norwegian Esther Program (The FK Health Exchange Program), Oslo: Scanteam

Hagen, L., Munkhondya, B. & Myhre, K., 2009. Similarities and mutual understanding: exchange experiences in Malawi for host and guest students. International nursing review, 56(4), pp.476–82.

Haglund, M.M. et al., 2011. Surgical capacity building in Uganda through twinning, technology, and training camps. World journal of surgery, 35(6), pp.1175–82

Haugland, M. et al., 2014. Factors for success in collaboration between high- and low-income countries: Developing a physiotherapy education programme in Sudan. European journal of physiotherapy, 16(3), pp.130–138.

Hopkins, J., Burns, E., & Eden, T. (2013). International twinning partnerships: An effective method of improving diagnosis, treatment and care for children with cancer in low-middle income countries. *Journal of Cancer Policy*, *1*(1-2), e8–e19.

James J, Minett C, Ollier L (2008). Evaluation of links between north and south healthcare organisations. DFID Health Resource Centre, 2008.

 Jones, F.A. et al., 2013. Do health partnerships with organisations in lower income countries benefit the UK partner? A review of the literature. Globalization and health, 9(1), p.38.

Kiernan P, O'Dempsey T, Kwalombota K, Elliott L, Cowan L (2014) “Evaluation of effect on skills of GP trainees taking time out of programme (OOP) in developing countries,” Education for Primary Care 25: 78-83; Radcliffe Publishing

Kinnear, J. a et al., 2013. A new partnership for anesthesia training in Zambia: reflections on the first year. Canadian journal of anaesthesia = Journal canadien d’anesthésie, 60(5), pp.484–91.

Leather, A.J.M. et al., 2010. International Health Links movement expands in the United Kingdom. International health, 2(3), pp.165–71.

Longstaff 2010 Innovative Workforce Development: the case for international health links, North East Strategic Health Authority

Parry, E.H.O. & Percy, D.B., 2007. Anaesthesia and hospital links: strengthening healthcare through South-North hospital partnerships. Anaesthesia, 62 Suppl 1, pp.15–20.

Paterson M, Telyukov A, 2007, Program Evaluation: AIHA Primary Healthcare Partnerships in the Newly Independent States (1998-2006)

Rao 2013.  New thinking on technical assistance to resolve knowledge and capacity gaps. HelpDesk Research Report, GSDRC Applied Knowledge Services (www.gsdrc.org).

Ritman, D. & Zegeye, H., 2012. The value of health partnerships: a practical approach to evidence. Tropical Doctor, 42(October), pp.241–242.

Roodebeke, E. de, 1994. Une voie d’avenir pour la cooperation: le partenariat hospitalier. Cahiers Sante, 4, pp.105–9.

Schönemann, Y & Weinmann, S, ESTHER Germany External Review 2010

Smith, C, 2013, Do UK health links improve health outcomes? Alma Mata Journal of Global Health, Sept 2013, 3

 Smith, C, 2012. The role of health links in international development: the need for greater evidence? *Tropical Doctor*, *42*(2), 65–66.

Smith, C, Pettigrew, L, Seo, H-N, Dorward, J, 2012. Combining general practice with international work: online survey of experiences of UK GPs. *JRSM short reports*, 3(7), p.46.

Syed, S. B., Dadwal, V., Rutter, P., Storr, J., Hightower, J. D., Gooden, R., Carlet, J., et al. (2012). Developed-developing country partnerships: benefits to developed countries? Globalization and health, 8, 17.

THET 2014 Health Partnership Scheme DFID Annual Report 2013/2014 submitted 23.6.14

Thomas K, Chowdhury J, Van Woerden H (2011) International Health Links: an investigation into health partnerships between Wales and Africa. Welsh Assembly Government.

Tierney, W.M. et al., 2013. “These are good problems to have…”: establishing a collaborative research partnership in East Africa. Journal of general internal medicine, 28 Suppl 3, pp.S625–38.

Wright, J., Walley, J., Philip, A., Petros, H., & Ford, H. (2010). Research into practice: 10 years of international public health partnership between the UK and Swaziland. *Journal of Public Health*, *32*(2), 277–282.

**Identified after completion of review**

Rutter, P., Syed, S B., Storr, J., Hightower, J D., Bagheri-Nejad, S., Kelley, E., Pittet, D. Development of an evaluation framework for African-European hospital patient safety partnerships. *BMJ Quality & Safety,* 2014 23:332-337

**Analysis by additional benefits referred to in literature and level of evidence**

|  | Level 1 | Level 2 | Level 3 | Level 4 | Level 5 |
| --- | --- | --- | --- | --- | --- |
| Sustainability |  | Baguley et al 2006  Corbin et al 2013  Haglund et al 2011  Doyle & Kelly 2013  Doyle & Kelly 2012  Thomas et al 2012  James et al 2008  Paterson & Telyukov 2007 |  |  |  |
| Ownership |  | Corbin et al 2013  Doyle & Kelly 2013  Doyle & Kelly 2012  Paterson & Telyukov 2007 |  |  |  |
| Flexibility |  | Corbin et al 2013  Doyle & Kelly 2013  Doyle & Kelly 2012  James et al 2008  Paterson & Telyukov 2007 |  |  |  |
| Mutual Benefit |  | Baguley et al 2006  Busse et al 2014  Longstaff 2012  THET 2014  Corbin et al 2013  Hagen et al 2009  Smith et al 2012  Doyle & Kelly 2013  Doyle & Kelly 2012  Thomas et al 2012  James et al 2008  Paterson & Telyukov 2007 |  |  | Jones 2013  Syed et al 2012 |
| Frontline |  | Doyle & Kelly 2013  Paterson & Telyukov 2007 |  |  |  |
| Peer-to-peer |  | Doyle & Kelly 2013  Doyle & Kelly 2012  Thomas et al 2012  Paterson & Telyukov 2007 |  |  |  |
| Institutional strengthening |  | Baguley et al 2006  Corbin et al 2013  Doyle & Kelly 2013  Doyle & Kelly 2012  Paterson & Telyukov 2007 |  |  |  |
| Value for money |  | THET 2014  James et al 2008  Paterson & Telyukov 2007 |  |  |  |
| Innovation |  | Baguley et al 2006  Doyle & Kelly 2013  Doyle & Kelly 2012  Paterson & Telyukov 2007 |  |  | Syed et al 2012 |
| Personal and Professional Development |  | Baguley et al 2006  Busse et al 2014  Longstaff 2012  THET 2014  Doyle & Kelly 2013  Doyle & Kelly 2012  James et al 2008  Paterson & Telyukov 2007 |  |  | Jones 2013  Syed et al 2012 |
| Knowledge and skills transfer |  | Busse et al 2014  Longstaff 2012  THET 2014  Corbin et al 2013  Haglund et al 2011  Smith et al 2012  Doyle & Kelly 2013  Doyle & Kelly 2012  James et al 2008  Paterson & Telyukov 2007 |  |  | Jones 2013  Syed et al 2012 |
| Reach less well funded areas of need |  | Corbin et al 2013  Doyle & Kelly 2013  Doyle & Kelly 2012  Paterson & Telyukov 2007 |  |  |  |
| Motivation |  | Longstaff 2012  Haarberg et al 2011  Smith et al 2012  Doyle & Kelly 2013  Doyle & Kelly 2012  James et al 2008  Paterson & Telyukov 2007 |  |  | Syed et al 2012 |
| Cultural competency |  | Busse et al 2014  Longstaff 2012  Corbin et al 2013  Hagen et al 2009  Doyle & Kelly 2013  Thomas et al 2012  James et al 2008  Paterson & Telyukov 2007 |  |  |  |
| Influencing policy |  | Doyle & Kelly 2013  Paterson & Telyukov 2007 |  |  |  |
